# ﻿Karyotype description and comparative chromosomal mapping of rDNA and U2 snDNA sequences in *Eigenmannialimbata* and *E.microstoma* (Teleostei, Gymnotiformes, Sternopygidae)

**DOI:** 10.3897/CompCytogen.v16i2.72190

**Published:** 2022-05-05

**Authors:** Cristian Andrés Araya-Jaime, Duílio Mazzoni Zerbinato de Andrade Silva, Luís Ricardo Ribeiro da Silva, Cristiano Neves do Nascimento, Claudio Oliveira, Fausto Foresti

**Affiliations:** 1 Instituto de Investigación Multidisciplinar en Ciencia y Tecnología, Universidad de La Serena, La Serena, Chile; 2 Departamento de Biología, Universidad de La Serena, La Serena, Chile; 3 Departamento de Biologia Estrutural e Funcional, Universidade Estadual Paulista Júlio de Mesquita Filho, Botucatu, SP, Brazil

**Keywords:** Electric fish, fish cytogenetics, freshwater fishes, karyotype evolution, repetitive DNA

## Abstract

The genus *Eigenmannia* Jordan et Evermann,1896 includes electric fishes endemic to the Neotropical region with extensive karyotype variability and occurrence of different sex chromosome systems, however, cytogenetic studies within this group are restricted to few species. Here, we describe the karyotypes of *Eigenmannialimbata* (Schreiner et Miranda Ribeiro, 1903) and *E.microstoma* (Reinhardt, 1852) and the chromosomal locations of 5S and 18S rDNAs (ribosomal RNA genes) and U2 snDNA (small nuclear RNA gene). Among them, 18S rDNA sites were situated in only one chromosomal pair in both species, and co-localized with 5S rDNA in *E.microstoma*. On the other hand, 5S rDNA and U2 snRNA sites were observed on several chromosomes, with variation in the number of sites between species under study. These two repetitive DNAs were observed co-localized in one chromosomal pair in *E.limbata* and in four pairs in *E.microstoma*. Our study shows a new case of association of these two types of repetitive DNA in the genome of Gymnotiformes.

## ﻿Introduction

The order Gymnotiformes is an endemic freshwater group inhabiting the Neotropical region and consisting of species capable of emitting low voltage continuous electric discharges (Alves-Gomes 2001; Lavoué et al. 2012). Among them, *Eigenmannia* is the most species-rich genus of the family Sternopygidae (Gymnotiformes), with 27 recognized species (Fricke et al. 2021). On the other hand, *Eigenmannia* is not a monophyletic assembly, and it is considered taxonomically ambiguous due to little morphological variation between species, which makes it difficult to define species-specific diagnostic characters (Alves-Gomes 1998; Albert 2001; Peixoto and Ohara 2019).

In recent years, cytogenetic studies in *Eigenmannia* were mainly limited to some species and / or karyomorphs (i.e., different karyotype forms), revealing a variable karyotype macrostructure, with diploid chromosome numbers ranging from (2n) = 28 to 46 chromosomes (Arai 2011; Silva et al. 2015b). In addition, different sex chromosome systems have been described, identifying standard systems such as XX/XY, ZZ/ZW in *E.virescens* (Almeida-Toledo et al. 2001; Henning et al. 2011; Fernandes et al. 2020), derived ZZ/Z0 system in E.propetrilineata (Araya-Jaime et al. 2017b), and multiple sex chromosome system X_1_X_1_X_2_X_2_/X_1_X_2_Y in *Eigenmannia* sp2 (Almeida-Toledo et al. 2000; Sene et al. 2014; Araya-Jaime et al. 2015), as well as species/karyomorphs without heteromorphic sex chromosomes (de Almeida Toledo et al. 1984; Almeida-Toledo et al. 2000, 2001; Silva et al. 2009; Henning et al. 2011).

The physical mapping of repetitive sequences in gymnotiform species has provided important data on the structure and organization of the genome that has allowed us to understand the processes of karyotypic evolution that these species have experienced, recognizing Robertsonian rearrangements as the most frequent mechanisms of chromosomal variability in Gymnotiformes (Milhomem et al. 2008; Giora and Fialho 2009; Nagamachi et al. 2010; da Silva et al. 2014; Utsunomia et al. 2014, 2018; Suárez et al. 2017; Rodrigues et al. 2021). The mapping of ribosomal DNA genes (18S rDNA and 5S rDNA) has been widely used in molecular cytogenetics of Gymnotiformes, where the evidence provided by several studies has made it possible to establish two distribution patterns of these sequences: i) 18S rDNA loci located on a single chromosome pair and ii) 5S rDNA sites located in multiple chromosomal pairs, which may be associated with transposable elements or U2 snDNA (small nuclear RNA gene) sequences (Scacchetti et al. 2011, 2012; Utsunomia et al. 2014; da Silva et al. 2016; Araya-Jaime et al. 2017b; Sochorová et al. 2018; Rodrigues et al. 2021).

Recently, the mapping of genes belonging to the U snDNA family increased the knowledge about the dynamics of tandemly repeated multigene families in vertebrates. This multigene family harbors genes coding for nine types of non-coding RNAs; namely U1, U2, U4, U4 atac, U5, U6, U6 atac, U11 and U12; which constitute a portion of the RNA-protein complex of the spliceosome (Valadkhan 2005; Matera and Wang 2014). In fish cytogenetics, the use of these repetitive markers is relatively recent, with data being reported for several groups, including Characiformes (Silva et al. 2015a; Santos et al. 2017; Serrano et al. 2017), Batrachoidiformes (Ubeda-Manzanaro et al. 2010), Cyprinodontiformes (Araya-Jaime et al. 2017a), Gadiformes (García-Souto et al. 2015), Perciformes (Xu et al. 2017), Cypriniformes (Sember et al. 2018), among others. In gymnotiform fish genomes, the cytogenetic reports of these sequences are restricted to U2 snDNA, recognizing two general chromosomal patterns: i) grouped in a single pair of chromosomes or ii) scattered throughout the genome and, in some cases, associated with 5S rDNA (Utsunomia et al. 2014; Araya-Jaime et al. 2017b). In this way, the U snDNA sequences represent a good repetitive marker to provide information on the evolutionary relations between closely related species, infer the homology between certain chromosomes present in different lineages, and trace the origin and evolution of specific chromosomes, in the context of the great karyotype diversity found among Gymnotiformes.

With the aim of expanding our knowledge about the chromosomal structure and the dynamics of repetitive DNA sequences in the *Eigenmannia* genome, we present for the first time the karyotype and chromosomal location of three repetitive DNA classes (18S and 5S rDNA and U2 snDNA) in *E.microstoma* and *E.limbata* from the Sao Francisco and the Amazon River basin, respectively. Our results show a new case of physical association between the 5S rDNA and U2 snDNA in Gymnotiformes.

## ﻿Material and methods

**Figure 1.**
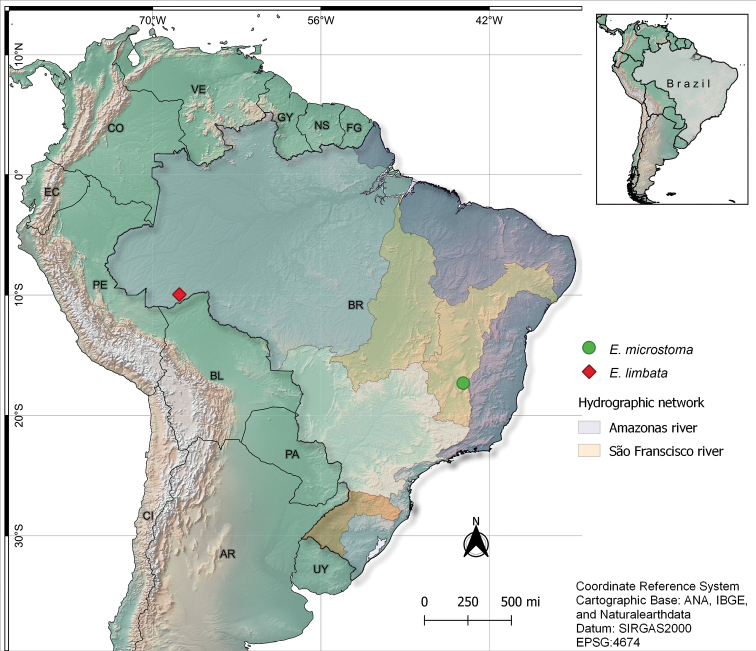
Location of *Eigenmannia* species in the Amazon and São Francisco basins.

Twelve individuals of *Eigenmannialimbata* and eight of *E.microstoma*, from the Amazon basin and the San Francisco River basin, respectively, were analyzed in this study (Fig. 1). After dissection, the specimens were fixed and preserved in 70% ethanol. Finally, these specimens were deposited in the fish collection of the Laboratório de Biologia e Genética de Peixes, UNESP, Botucatu-SP. The animals were collected in accordance with Brazilian environmental protection legislation (Collection Permission MMA/IBAMA/SISBIO-number 3245) and the procedures for fish sampling, maintenance and analysis were performed in compliance with the Brazilian College of Animal Experimentation (**COBEA**) and approved (protocol 504) by the Bioscience Institute/Unesp Ethics Committee on the use of Animals (**CEUA**).

Mitotic chromosomes were obtained by direct preparation from the cephalic kidney according to Foresti et al. (1993), and slides for conventional analysis were stained with 5% Giemsa solution in a phosphate buffer at pH 6.8. The constitutive het­erochromatin (CH) was detected following Sumner (1972). Images were captured with a digital camera (Olympus DP90) in the Olympus BX6 epifluorescence photomicroscope and acquired using cellSens Dimension (Olympus, Sapporo-Japan). Image treatment, optimization of brightness and contrast was performed using the Adobe Photoshop CS6 program. The arm ratio (Levan et al. 1964) was used to classify the chromosomes as metacentric (m), submetacentric (sm), subtelocentric (st), and acrocentric (a). For counting the total number of chromosome arms or fundamental number (NF), chromosomes m, sm, st were considered bi-armed, while acrocentric chromosomes (or indistinguishable st/a) were classified as mono-armed chromosomes.

Fluorescence *in situ* hybridization (FISH) procedure was performed according to Pinkel et al. (1986). The 18S, 5S rDNA and U2 snRNA gene probes were obtained from the genomic DNA of *E.microstoma* which was extracted using Wizard Genomic DNA Purification Kit (PROMEGA, Madison, Wisconsin, USA). The rDNA probes were amplified by polymerase chain reaction (PCR), using the primers 18SF (5’ CCGCTTTGGTGACTCTTGAT 3’) and 18SR (5’ CCGAGGACCTCACTAAACCA 3’) (White et al. 1990), 5SF (5’ TACGCCCGA TCTCGTCCGATC 3’) and 5SR (5’ CAGGCTGGTATGGCCGTAACG 3’) (Pendas et al. 1994) and U2F (5’ ATCGCTTCTCGGCCTTATG 3’) and U2R (5’ TCCCGGCGGTACTGCAATA 3’) (Bueno et al. 2013). PCR products were verified in 1% agarose gel. 18S rDNA probe (600 pb long fragment) were labeled with biotin-14-dATP (Dig Nick Translation mix, Roche, Applied Science, Penzberg, Germany), while the U2 snRNA gene probe (150 bp) was labeled by PCR with biotin-16-dUTP (Roche). Hybridization signals were detected using FITC–avidin (conjugated fluorescein isothiocyanate–avidin; Sigma-Aldrich, St Louis, MO, USA). 5S rDNA probe (300 pb) was labeled with digoxigenin-11-dUTP (Biotin Nick Translation mix, Roche) and the hybridization signals were detected using anti-digoxigenin-rhodamine (Roche). The chromosomes were counterstained with 0.2 μg/mL of 4’, 6-diamidino-2-phenylindole (DAPI) in the Vectashield mounting medium (Vector, Burlingame, CA).

## ﻿Results

The diploid chromosome number (2n) of the *E.microstoma* was 38 chromosomes, with a karyotype composed of 8m + 10sm + 20a chromosomes (NF = 56), while *E.limbata* had 2n = 38 and karyotype composed of 8m + 4sm + 26a chromosomes (NF = 50). Morphologically differentiated sex chromosomes were not found in either species (Table 1).

**Table 1.** Cytogenetic features and collection sites of *Eigenmannia* species.

Species (N)	2n	Karyotype formula	Sample localities	Hydrographic basin	Coordinates (DDM)
*E.limbata* (♂7, ♀5)	38	8m+4sm+26a	Rio Branco-AC	Amazonas	9°57'27.10"S, 67°46'55.40"W
*E.microstoma* (♂5, ♀3)	38	8m+10sm+20a	Francisco Dumont-MG	São Francisco	17°18'57.80"S, 44°10'23.00"W

**Figure 2.**
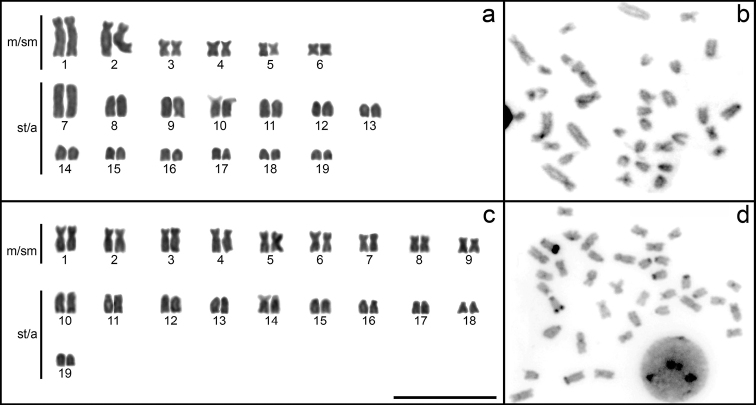
Chromosomes stained with Giemsa and C-banded **a, b** karyotype and C-banded metaphase of *E.limbata***c, d** karyotype and C-banded metaphase of *E.microstoma*. Scale bar: 10 μm.

C-banding technique revealed significant differences in the patterns of CH distribution between the analyzed species. Both species displayed pericentromeric regions of CH in all chromosomes and *E.microstoma* possessed additional interstitial blocks on several chromosomes (Fig. 2).

**Figure 3.**
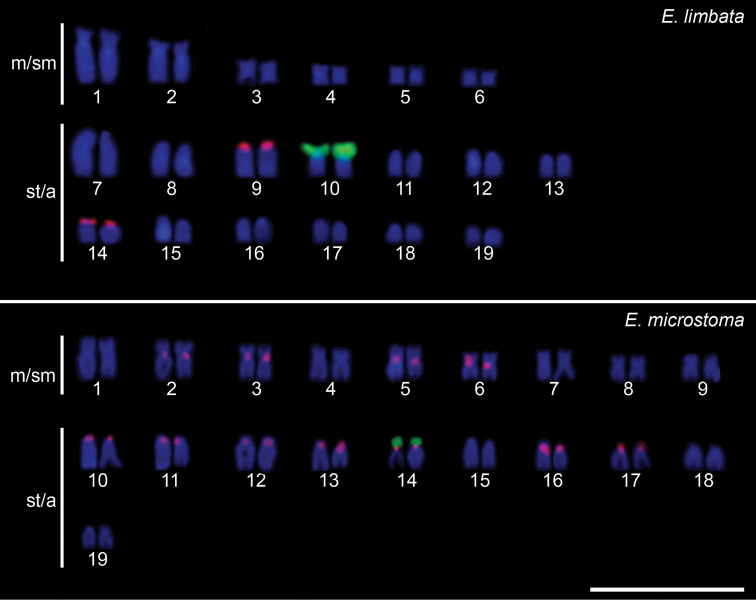
Karyotypes of *Eigenmannia* species after FISH with 5S (red) and 18S (green) ribosomal DNA probes and counterstained with DAPI. Scale bar: 10 μm.

The 18S rDNA site was located by FISH in a single chromosomal pair in both species, namely pair No. 10 in *E.limbata* and pair No. 14 in *E.microstoma* (Fig. 3). The 5S rDNA sites showed a considerable variation in the number and locations in analyzed species. These sites were detected in two chromosomal pairs in *E.limbata* and in 11 chromosomal pairs in *E.microstoma* (Fig. 3).

**Figure 4.**
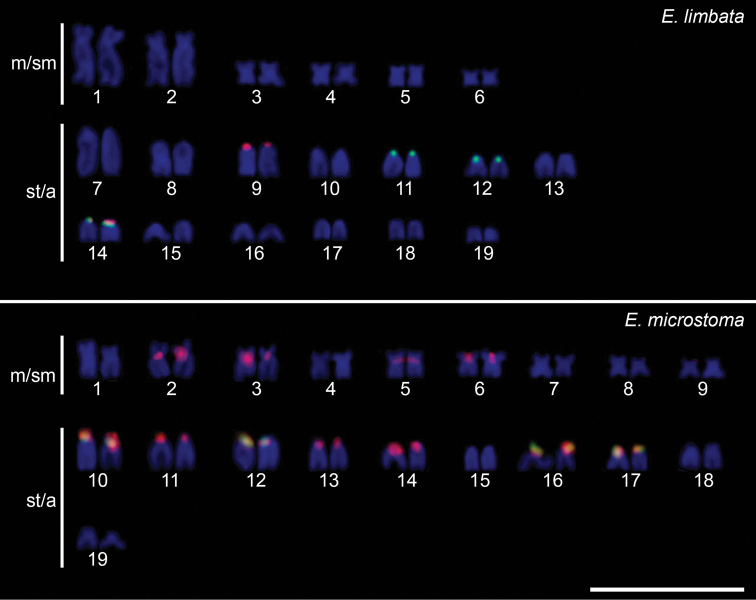
Karyotypes of *Eigenmannia* species after FISH with 5S rDNA (red) and U2 snDNA (green) probes and counterstained with DAPI. Note that, the two repetitive DNAs are located adjacently on the same pair (14) in *E.limbata* and they are located adjacently on four chromosome pairs (10, 12, 16 and 17) in *E.microstoma*. Scale bar: 10 μm.

**Figure 5.**
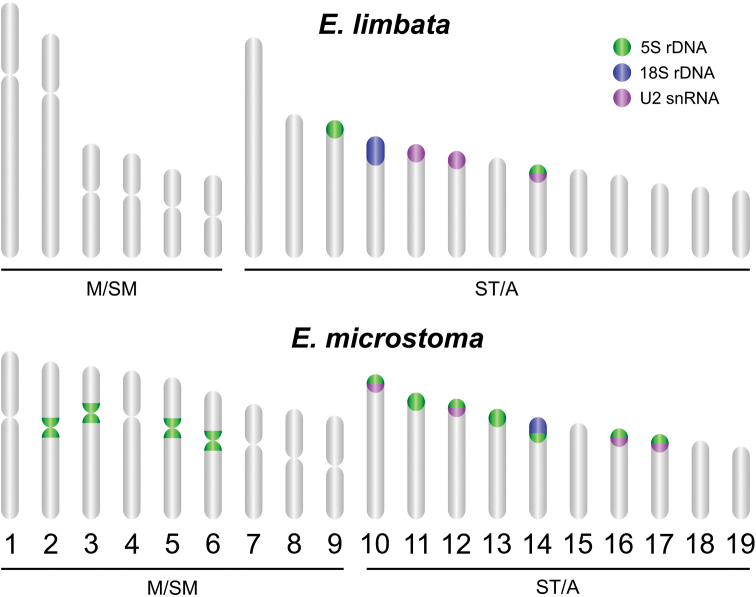
Idiogram of *Eigenmannia* species showing the location of repetitive DNAs.

The distribution of the U2 snDNA sites was variable in terms of the number, chromosomal location, and number of co-localized sites with 5S rDNA between species. U2 snDNA sites were placed on three chromosomal pairs (11, 12 and 14) in *E.limbata* and in the chromosome pairs Nos 10, 12, 16 and 17 in *E.microstoma* (Fig. 4). These sites were co-localized with the 5S rDNA sites in the pair No. 14 in *E.limbata* and in all pairs in *E.microstoma* (Fig. 4). The location of all repetitive DNAs mapped by FISH is summarized in the ideogram presented in Fig. 5.

## ﻿Discussion

The species *E.limbata* and *E.microstoma* were analyzed cytogenetically for the first time, showing the same 2n (38 chromosomes), but different NF and karyotypic structure (Table 1). Previous cytogenetic studies in *Eigenmannia* have consistently reported this same 2n (38 chromosomes), but there are wide variations in terms of the reported karyotypic formula, NF and sex chromosome system (Almeida Toledo et al. 1984; Moysés et al. 2010; Henning et al. 2011; de Sene et al. 2014; Araya-Jaime et al. 2017b; Fernandes et al. 2020). These differences in karyotypic structure and NF can be explained by the occurrence of Robertsonian rearrangements, which may be participating as an important postzygotic reproductive isolation mechanism in *Eigenmannia.* This circumstance could be related to their low population sizes and low mobility, which would facilitate the fixation of chromosomal polymorphisms (Moysés et al. 2005, 2010; Giora and Fialho 2009; Silva et al. 2009, 2015b).

A single chromosome pair carrying the NOR has been reported for most of the species of the Sternopygidae family, although the chromosomal location of the NOR varies between species and populations; therefore, a simple NOR phenotype can be an ancestral feature in the genome of Sternopygidae (de Almeida-Toledo et al. 2001; dos Santos Silva et al. 2008; de Sene et al. 2014; Araya-Jaime et al. 2017b; Fernandes et al. 2020; Rodrigues et al. 2021). However, within Gymnotidae, the case of *Gymonotuscoatesi* is reported as the only representative of this family with multiple 18S rDNA sites (Machado et al. 2017). Furthermore, a considerable variability has been observed in the size of the NOR region within Sternopygidae (dos Santos Silva et al. 2008; de Sene et al. 2014; Silva et al. 2015b; Fernandes et al. 2017; Rodrigues et al. 2021). Accordingly, we observed this NOR heteromorphism between *E.limbata* and *E.microstoma*, in which the NOR region of *E.limbata* is considerably larger than that of *E.microstoma* (Fig. 3). This could be a consequence of tandem duplication of ribosomal genes which could form through several mechanisms including unequal exchange of sister chromatids or unequal crossing over during meiosis (Charlesworth et al. 1994; Eickbush and Eickbush 2007; Bianciardi et al. 2012).

On the other hand, multiple 5S rDNA sites observed in *E.limbata* and *E.microstoma* (Fig. 3) appear to be a widely recognized feature within Gymnotiformes, with evidence in representatives of *Gymnotus* (da Silva et al. 2011; Scacchetti et al. 2011, 2012; Utsunomia et al. 2014; da Silva et al. 2016, 2019), *Eigenmannia* (de Sene et al. 2014; Araya-Jaime et al. 2017b; Fernandes et al. 2020), *Sternopygus* (Fernandes et al. 2017) and *Archolaemus* (Rodrigues et al. 2021). Ribosomal DNA sites are considered as hot spots for chromosomal rearrangements due to their organization into long stretches of conserved tandemly repeated sequences and their high transcription activity, which means they are susceptible to chromosomal breakage and/or non-allelic homologous recombination, increasing thus the probability of occurrence of chromosomal rearrangements, such as fusions, fissions and inversions (Rosa et al. 2012; Barros et al. 2017; Potapova and Gerton 2019; Warmerdam and Wolthuis 2019; Deon et al. 2020). Furthermore, the rDNA dynamics has been also correlated with the insertion of transposable elements, or other repetitive DNAs, into non-transcribed spacers (NTS) of 5S rDNA units, as has been observed in the genomes of *G.inaequilabiatus* (Scacchetti et al. 2012), *G.paraguensis* (da Silva et al. 2011) and *G.mamiraua* (da Silva et al. 2016). Thus, both mentioned mechanisms could explain the chromosomal dynamics of these sequences in gymnotiform genomes (de Sene et al. 2014; da Silva et al. 2016; Araya-Jaime et al. 2017b; Fernandes et al. 2017). In our case, given that 5S rDNA probe was prepared from the genomic DNA of *E.microstomata* in which we then revealed 22 signals, and that only four signals were evidenced in *E.limbata*, a possible explanation may be that the 300 bp long 5S rDNA fragment contains inserts of other repeats in its NTS region which might have promoted spreading of 5S rDNA clusters and/or generated additional non-5S rDNA signals in *E.microstomata*. In that case, only four signals in *E.limbata* might mean that the signal pattern is much less affected by the action and/or additional accumulation of the associated repeat(s). Although a single consistent PCR amplification product was obtained to be a template for the FISH probe preparation, thereby evidencing a lack of detectable amounts of 5S rDNA sequence variants or truncated copies, we cannot directly evaluate the possible presence and contribution of other repeats as we did not sequence the 5S rDNA fragment and consequently weren’t looking for admixed repetitive sequences. We may, however, conclude that the chromosomal behavior of the 5S rDNA sites observed in this work is congruent with the patterns previously reported for *Eigenmannia*, such as the number of variable sites and their association with 18S rDNA and U2 snDNA clusters (de Sene et al. 2014; Araya-Jaime et al. 2017b).

The results presented here, for *E.limbata* and *E.microstoma*, represent the first case, within *Eigenmannia*, of multiple sites for U2 snDNA (Fig. 4), highlighting in *E.microstoma* the presence of three chromosomal pairs carrying U2 snDNA sites, where one of them (pair No 14) is co-localized with 5S rDNA, while in *E.limbata*, the four chromosomal pairs carrying U2 snRNA genes are co-localized with 5S rDNA. The previous report by Araya-Jaime et al. (2017b) described the karyotype of E.afftrilineata with a single U2 snDNA site being co-localized with 5S rDNA. These results reinforce the dynamic nature of these sequences and show that the 5S rDNA / U2 snRNA association would be a characteristic feature of the *Eigenmannia* genome. In other Gymnotiformes, six *Gymnotus* species are reported with a single U2 snRNA carrier pair, while only in *G.pantal* and *Archolaemusjanae*, multiple sites for U2 snRNA have been reported (Utsunomia et al. 2014; Rodrigues et al. 2021). None of the species mentioned above exhibits co-localization between U2 snDNA with other sequences been reported.

## ﻿Conclusion

In the present work, the cytogenetic analysis carried out in the species *E.limbata* and *E.microstoma* reinforced the chromosomal variability reported for the genus, evidencing the occurrence of notable differences between the karyotypes of the species / karyomorphs studied up to here, even though the 2n mostly observed is 2n = 38 chromosomes. The chromosomal location of the 5S and 18S rDNA clusters observed in the species studied here followed the same pattern observed in Gymnotiformes with a single NOR-bearing pair and multiple sites for 5S rDNA. On the other hand, the dynamic nature of the U2 snRNA sites stands out, together with the co-localization with 5S rDNA genes, as a characteristic feature of the *Eigenmannia* genome. Finally, the results presented here reinforce the postulate that cytogenetic features (conventional and molecular) could be considered as important markers for taxonomic diagnosis and for the description and characterization of the existing biodiversity in Gymnotiformes.

## ﻿Author’s contribution

Conceptualization: CAJ, CO, FF; experimental design: CAJ, LRRS, FF; collected samples: CAJ, DMZAS, LRRS, CNN; cytogenetics analyses: CAJ, DMZAS, LRRS, CNN, FF; contributed with reagents/materials/analysis tools: DMZAS, CO, FF. All authors wrote, read, and approved the manuscript.
